# Extract from a Paper on the Treatment of Varicose Veins by the Needle and Twisted Suture

**Published:** 1842-04

**Authors:** T. B. Peacock

**Affiliations:** Late House Surgeon to the Chester Infirmary, &c.


*Extract from a paper on the Treatment of Varicose Veins by the needle and twisted suture.* By T. B. Peacock, Esq., late House Surgeon to the Chester Infirmary, &c.—I was first led to make trial of this plan from reading the report of a case by Mr. Melvin, in the number of the London Medical Gazette for July 7th, 1838, and I have since applied it myself, or seen it made use of by the surgeons to the Chester Infirmary, in at least thirty cases, of several of the most important of which I have retained notes. The plan adopted has been that recommended in the paper referred to, of passing a common curved suture needle under the vein, constricting it with a thread in the figure-of-8 form, and having turned the needle on its side, retaining it there by straps of adhesive plaster: at the end of two or three days, the ligature, if only moderately tightened at first, will require to have a fresh one passed over it; and in two or three more the needle may be removed. Several different methods have been proposed for effecting the obliteration of the vein by the needle; but this, which was originally introduced by Velpeau, as being the most simple, is that which I have always adopted. The length of time which it will be necessary for the needle to remain will depend on whether it is intended simply to excite suppuration, or to ulcerate out; the last being the course which I have usually followed, as in one or two instances, in which the needle was withdrawn after exciting suppuration, the obliteration of the vein was found not to have been effected. This plan has, however, been objected to as leaving a sore difficult to heal afterwards; but in only one instance have I seen it attended by any such result. For the needle to ulcerate its way out, the time usually required will be from a week to ten days; but it will vary greatly according to the state of the part in which it is applied: in the immediate neighborhood of an ulcer, where the skin is thin and inflamed, a day or two will often suffice to commence the ulcerative action, and three or four for the needle to escape; while, when inserted some distance from the seat of the disease, and beneath sound integument, the process will require ten days, a fortnight, or even longer. Thus, in a case lately under my charge, where the needle was inserted beneath a tender sinus on the instep, leading to a small ulcer about an inch above, it ulcerated out in three days; while at the same time, in another case, a needle was placed under each saphæna, and one beneath the common vein, at their point of union; the needle on the anterior branch was not removed till the twelfth day, and the other two not till the nineteenth. I have since seen two instances in which the needles were retained till the end of the third week. Generally speaking, when inserted over a bone, they excite ulceration more rapidly than when upon soft parts; and I am inclined to think that, in the last situation, they are more apt to give rise to an undue degree of inflammation: at least, in the only two cases in which their application was followed by troublesome abscesses, they had been inserted beneath sinuses in the calf of the leg. Considerable pain is sometimes excited by the operation, but it usually soon subsides; and I have not, in any instance, known tenderness to extend in the course of the vein above two or three inches from the point of constriction; and in none has it resisted ordinary treatment: indeed, in no instance which I have seen, have any serious symptoms resulted from the operation.

The cases in which I have found this treatment applied, have been in small irritable sores remaining after the bursting of large varicose sinuses, inveterate ulcers connected with a generally enlarged condition of the veins of the limb, and œdema of the leg and ankle, either simple or attended with a serous discharge from the skin; and in all of the cases but two in which I have seen it had recourse to, the results have been most satisfactory; and in these, as only one needle was inserted, and other sinuses were left unobliterated, success was hardly to be expected. The number of needles which I have generally seen inserted has been three or four in each limb, but, in some instances, five or six have been applied; the rule adopted having been generally to insert in a case of varicose ulcer one under each enlarged vein, an inch or so below the ulcer, and again on each trunk a few inches above it, selecting for the points of their insertion the largest sinuses. Sometimes I have adopted the plan mentioned by Mr. Dodd, of placing on each vein two needles an inch or an inch and a half apart, so as to effect adhesion of the sides of the intervening tract; and in these cases the main trunk will, after the cure is effected, be often found contracted to a firm cord up to the point at which the next large vein communicates with it; while, where a single needle only is inserted, the portion of the sinuses around is often not affected by the operation.

The effect produced on the sore by the obstruction to the course of the large veins in connection with it, is often most rapid; the inflamed margin gradually subsides, the edges become depressed, granulations spring up, and cicatrization quickly proceeds; and sores which have been liable to bleed entirely lose that tendency, the granulations becoming firm. I have, however, observed what has been noticed before by Mr. Dodd, that the healing process was not equally rapid throughout, the good effect produced by the needles sometimes gradually subsiding, and considerable difficulty being experienced in obtaining the entire healing of the sore.

In this way ulcers which had long been under treatment, without deriving any advantage, have, in several instances, been cured, and others which were found to return as soon as the patient resumed his work, have, by the aid of a laced stocking, been kept healed; indeed, not only does it appear to be a rapid method of effecting the cure of these cases, but I

am inclined to regard it also as a more permanent one. The first case in which I made trial of the practice was one of œdema of both legs, attended with excoriation of the skin, and a fœtid discharge, connected with a very varicose state of the large veins. The man, by trade a rope-maker, had been repeatedly under treatment before with very partial benefit; and no sooner did he resume work than the disease returned. On this occasion he had been subjected to the ordinary treatment during a month that he had resided in the infirmary, but with little or no advantage. Under these circumstances, as the case seemed to offer a fair opportunity for treatment with the needles, three were inserted beneath large sinuses in one leg, which was nearly well before the same plan was adopted in the other. He was discharged, entirely cured, on needles being introduced in the other limb, in six weeks from the commencement of the treatment. Two years have now elapsed, and he continues perfectly free from any return of his complaint.—Of two men one had suffered from varicose ulcers on both legs for nine years, the other for five; and both had been several times under treatment in neighboring infirmaries, but no sooner did they return to their work, that of cotton-spinning, than the ulcers again broke out. Seven needles were inserted in the legs of one, and three in the other, and both were cured, one in seven, the other in three weeks, and continued so for at least four months, during which I had an opportunity of noticing them. Indeed the absence of any pain, swelling, or weakness in the limbs, which they said, as healed before, they had always found to continue, and the sound appearance of the cicatrices, afforded a fair prospect of permanent cures having been effected. The state of the limb afterwards, and the pale, healthy-looking cicatrices, form a great contrast between cases treated by this and by the ordinary methods. I had a case recently under my charge, in which an ulcer, fully the size of the palm of the hand, was entirely cured in little more than a month, and this notwithstanding that copious suppuration was excited by the needles in the cellular membrane of the calf of the leg. This patient had previously been subjected to treatment for four months, with every advantage of circumstances for the cure of a sore in the same situation; and the case was further interesting as being attended by severe pain in the sole of the foot—an occurrence which was met with in one of Mr. Dodd's patients—and having been an old man of 70; while Bonnet, in an essay on this subject, published in Paris, has stated that the operation will not be successful after the age of 60, in consequence of the indisposition of the blood to coagulate, and that it should not be attempted. I heard of the man several months after his discharge; he was following his work and his limb continued sound. I regret that, in consequence of most of the patients on whom the plan was tried in the infirmary residing at a distance, I am not able to speak of them after they left the institution.

The above remarks were written more than twelve months ago. I have now nothing further to add than that additional experience fully confirms the opinion expressed of the safety and rapidity of the cure of disease dependent on varicose veins, by the plan referred to, and I have reason to regard it as also a permanent one, care being of course taken to support the limb by a laced stocking or bandage, as otherwise the same cause which gave rise to the varicose condition of the veins will lead to the dilatation of fresh ones.—*Philadelphia Examiner*, from *London Med. Gaz.*, *Nov.* 5, 1841.

